# Contact Lens Wearer Demographics and Risk Behaviors for Contact Lens-Related Eye Infections — United States, 2014

**DOI:** 10.15585/mmwr.mm6432a2

**Published:** 2015-08-21

**Authors:** Jennifer R. Cope, Sarah A. Collier, Maya M. Rao, Robin Chalmers, G. Lynn Mitchell, Kathryn Richdale, Heidi Wagner, Beth T. Kinoshita, Dawn Y. Lam, Luigina Sorbara, Aaron Zimmerman, Jonathan S. Yoder, Michael J. Beach

**Affiliations:** 1 Division of Foodborne, Waterborne, and Environmental Diseases, National Center for Emerging and Zoonotic Infectious Diseases, CDC; 2 Contact Lens Assessment in Youth (CLAY); 3 Clinical Trial Consultant, Atlanta, Georgia; 4 College of Optometry, The Ohio State University, Columbus, Ohio; 5 College of Optometry, State University of New York, New York, New York; 6 College of Optometry, Pacific University, Forest Grove, Oregon; 7 Southern California College of Optometry at Marshall B. Ketchum University, Fullerton, California; 8 School of Optometry and Vision Science, University of Waterloo, Waterloo, Ontario, Canada

Contact lenses provide safe and effective vision correction for many Americans. However, contact lens wearers risk infection if they fail to wear, clean, disinfect, and store their contact lenses as directed. Over the past decade, CDC has investigated several multistate outbreaks of serious eye infections among contact lens wearers, including *Acanthamoeba* keratitis (1). Each investigation identified frequent contact lens hygiene-related risk behaviors among patients. To guide prevention efforts, a population-based survey was used to estimate the number of contact lens wearers aged ≥18 years in the United States. A separate online survey of contact lens wearers assessed the prevalence of contact lens hygiene-related risk behaviors. Approximately 99% of wearers reported at least one contact lens hygiene risk behavior. Nearly one third of contact lens wearers reported having experienced a previous contact lens-related red or painful eye requiring a doctor’s visit. An estimated 40.9 million U.S. adults wear contact lenses, and many could be at risk for serious eye infections because of poor contact lens wear and care behaviors. These findings have informed the creation of targeted prevention messages aimed at contact lens wearers such as keeping all water away from contact lenses, discarding used disinfecting solution from the case and cleaning with fresh solution each day, and replacing their contact lens case every 3 months.

Nearly one million U.S. health care visits for keratitis (inflammation of the cornea) or contact lens complications occur annually, at a cost of $175 million (2). The largest single risk factor for microbial keratitis is contact lens wear (3). Quantifying the number of contact lens wearers at risk for serious eye infections is important for future prevention efforts, but requires a population-based estimate of the number of contact lens wearers in the United States.

To estimate the size of the population at risk for contact lens-related complications in the United States and describe its demographics, the Porter Novelli 2014 summer ConsumerStyles survey, an online survey of 4,269 respondents, was used.* Participants in the ConsumerStyles survey were part of market research firm GfK’s Knowledge Panel. Panel members are recruited using address-based probability sampling methods and are provided with internet access and a computer if needed. ConsumerStyles survey participants receive entry into a monthly sweepstakes with a prize usually worth <$500. Statistical weighting was used to make the panel representative of the U.S. population on age, sex, race/ethnicity, education level, household income, household size, census region, metropolitan status, and internet access before joining the panel. Respondents were asked demographic questions and what type of contact lenses they wore.

To describe the prevalence of contact lens hygiene-related risk behaviors, an adapted version of the Contact Lens Risk Survey, a previously validated survey,† was administered to a convenience sample of online, contact lens-wearing panelists to describe the prevalence of usual contact lens hygiene-related risk behaviors. Participants were members of market research firm Schlesinger Associates’ research panel and wore contact lenses. Panel members are recruited in-person or via internet advertising, email campaigns, or telephone calls. Questions about usual contact lens-related behaviors included the following responses regarding the usual frequency of the behavior: always, fairly often, sometimes, infrequently, or never. For this report, questions with these responses were coded as “ever” if the response was not “never.”

Using the population-based survey, an estimated 40.9 million persons in the United States aged ≥18 years wear contact lenses (16.7% of U.S. adults)§; 93.0% of contact lens wearers reported wearing soft contact lenses (lenses made of soft, flexible plastics that allow oxygen to pass through to the cornea). Overall, contact lens wearers were younger, female, more educated, and of white, non-Hispanic race/ethnicity when compared with non-contact lens wearers (Table 1). No significant geographic differences between contact lens wearers and non-contact lens wearers were found. Among subtypes of contact lens wearers, rigid contact lens (lenses made of more durable materials resistant to deposit buildup) wearers did not differ significantly in age from non-contact lens wearers, although wearers of soft, daily disposable (lenses worn once and discarded) and overnight contact lens (lenses prescribed for wear while sleeping) were significantly younger.

Approximately 1,000 contact lens wearers completed the Contact Lens Risk Survey. Respondents were mostly female (82%) and aged ≥40 years (62%). Approximately 99% of respondents reported at least one contact lens hygiene behavior previously associated with an increased risk for eye infection or inflammation (Table 2). Half or more of wearers reported ever sleeping overnight in contact lenses (50.2%), ever napping in contact lenses (87.1%), ever topping off disinfecting solution (adding new solution to existing solution in the contact lens case instead of emptying and cleaning the case before adding new solution, 55.1%), extending the recommended replacement frequency of lenses (49.9%) or cases (82.3%), and ever showering (84.9%) or swimming (61.0%) in contact lenses. Approximately one third (35.5%) of contact lens wearers reported ever rinsing their lenses in tap water and 16.8% reported ever storing their lenses in tap water. Almost all rigid wearers (91.3%) reported ever rinsing their lenses in water, and 33.3% reported ever storing their lenses in tap water. Nearly one third of all wearers reported ever having experienced a contact lens-related red or painful eye that required a doctor’s visit.

## Discussion

An estimated one in six adults in the United States wears contact lenses, and one third of them report at least one health care visit for a red or painful eye while wearing lenses. Approximately 99% of contact lens wearers reported at least one risk behavior ever for eye infections or inflammation. Of particular concern, contact lens wearers of all types frequently reported exposure of their contact lenses to water, including storing or rinsing their lenses in tap water and showering or swimming while wearing lenses. Exposure of lenses to water raises the risk for infection because microorganisms living in water can be transferred to the eye. Even household tap water, although treated to be safe for drinking, is not sterile and contains microorganisms that can contaminate lens cases and contact lenses and cause eye infections.

Sleeping in contact lenses was a frequently reported behavior. Although many soft and some rigid contact lenses have U.S. Food and Drug Administration-approved indications for overnight wear, sleeping in any type of contact lens increases risk for eye infection, although the precise mechanism is not known (4). Noncompliance with recommended lens and case replacement schedules was also commonly reported. Infrequent replacement of contact lens cases has been linked to serious eye infections (5). Additionally, contact lens wearers who do not follow recommended contact lens replacement schedules have more complications and eye discomfort (6). These behaviors raise the risk for eye infections because repeated handling of the lens and case provides opportunities for introduction of microorganisms, while the moist surface of the lens and case provide an environment conducive to microbial growth. This risk is compounded if wearers top off solution in the case, as a majority of surveyed contact lens wearers reported having done at least once. Topping off also decreases the effectiveness of contact lens disinfection (7).

Daily disposable contact lens wearers might have a lower risk for infection if contact lenses are disposed of daily as recommended. Although 40% of daily disposable contact lens wearers did not use a case, thereby avoiding potential contamination associated with the case, a large proportion of daily disposable contact lens wearers did use a case and did so improperly, using tap water to store their lenses.

The number of contact lens wearers in the United States presented here is higher than previous estimates. Another study estimated 38 million contact lens wearers, although the data collection methods were not described (8). A more recent study used data from the National Health and Nutrition Examination Survey (NHANES) and estimated that 18.6 million persons aged ≥12 years wore contact lenses (9). However, the NHANES protocol used a more restrictive contact lens wearer definition¶ and might have underestimated the total number of contact lens wearers in the United States. The demographic patterns observed in the population used for the estimate reported here were similar to the NHANES population; however, the estimate reported here, based on self-reported contact lens use, is a more inclusive estimate. Contact lens wearers are younger on average than non-contact lens wearers. Teens and college age persons (those aged 15–25 years) have been associated with lower contact lens compliance and with higher risk for corneal inflammatory events, a category of eye problems that includes serious eye infections (10).

The findings in this report are subject to at least two limitations. First, the estimated number of contact lens wearers in the United States reported here does not include those aged <18 years. Since younger age is a predictor of more frequent complications, the current estimate does not include some contact lens wearers who might be most at risk for complications. Second, the Contact Lens Risk Survey used a convenience sample and respondents were more likely to be older and female than the general contact lens-wearing population. Because risk factors have been shown to vary by age, the survey might have underestimated the prevalence of contact lens risk behaviors.

**Summary**

What is already known on this topic?

Contact lenses are a safe and effective form of vision correction for the millions of Americans who require it, if worn and cared for as directed. Poor contact lens hygiene behaviors such as exposing contact lenses to water and topping off storage cases with disinfection solution put contact lens wearers at risk for eye infections.

What is added by this report?

In 2014, there were an estimated 40.9 million contact lens wearers aged ≥18 years in the United States. Approximately 99% of contact lens wearers completing the Contact Lens Risk Survey in 2014 reported at least one contact lens hygiene behavior ever that could put them at risk for an eye infection. One third of contact lens wearers reported ever experiencing a red or painful eye that required a doctor’s visit.

What are the implications for public health practice?

Prevention efforts could include vigorous health promotion activities that encourage contact lens wearers to improve their hygiene behaviors, such as keeping all water away from contact lenses, discarding used disinfecting solution from the case and cleaning with fresh solution each day, and replacing their contact lens case every 3 months.

Tens of millions of U.S. adults enjoy the benefits of contact lens wear, but many of them might be increasing their risk for complications because of poor wear and care behaviors. Improved estimates of the extent of contact lens-associated disease and increased surveillance capacity for microbial keratitis are needed. Prevention efforts could include vigorous health promotion activities that encourage contact lens wearers to improve their hygiene behaviors, such as keeping all water away from contact lenses, discarding used disinfecting solution from the case and cleaning with fresh solution each day, and replacing their contact lens case every 3 months (Box).

BOX **Wear and care recommendations to reduce the risk for contact lens-associated complications** * †

**Table:** 

**Contact lens habits and hygiene** Never sleep in contact lenses unless advised to do so by an eye care provider. Keep all water away from contact lenses. Avoid showering while wearing contact lenses, remove them before using a hot tub or swimming, and never rinse or store contact lenses in water. **Contact lenses and supplies** Replace contact lenses as often as recommended by an eye care provider. Discard used solution from the contact lens case and clean it with fresh solution, never water, every day. Store contact lens case upside down with the caps off after each use. Replace the contact lens case at least once every 3 months. **Eye care provider involvement** Visit an eye care provider as often as recommended by your primary health care provider. Remove contact lenses immediately and call an eye care provider if you are experiencing eye pain, discomfort, redness, or blurred vision. **Be prepared** Carry a backup pair of glasses with a current prescription in case contact lenses need to be removed. Additional information about healthy contact lens wear and care is available at http://www.cdc.gov/contactlenses and http://www.cdc.gov/contactlenses/show-me-the-science.html.

## Figures and Tables

**TABLE 1:** Demographic characteristics of wearers and non-wearers of contact lenses, by type of contact lens — United States, 2014*

Characteristic	Non-wearers (n = 3,528)		Contact lens wearer, by type										All contact lenses (n = 709)	

			Daily disposables (n = 82)		Planned replacement, soft (n = 461)		Overnight, soft (n = 55)		Rigid (n = 46)		Other† (n = 65)			
						
	(%)	(95% CI)	(%)	(95% CI)	(%)	(95% CI)	(%)	(95% CI)	(%)	(95% CI)	(%)	(95% CI)	(%)	(95% CI)
**Age group (yrs)**														
18–25	(11.1)	(9.6–12.6)	(19.5)	(7.3–31.6)	(20.7)	(15.4–26.0)	(16.5)	(2.1–30.9)	(13.7)	(0.0–30.8)	(11.9)	(0.5–23.3)	(19.0)	(14.8–23.2)
25–29	(7.3)	(6.1–8.5)	(7.9)	(0.0–17.2)	(12.6)	(8.6–16.6)	(19.5)	(3.1–35.9)	(15.8)	(1.4–30.1)	(15.8)	(2.0–29.6)	(13.1)	(9.6–16.5)
30–39	(15.4)	(13.8–16.9)	(22.5)	(11.9–33.0)	(26.1)	(20.9–31.4)	(37.0)	(18.7–55.2)	(8.2)	(0.0–17.4)	(19.9)	(6.7–33.1)	(24.8)	(20.7–29.0)
40–49	(15.7)	(14.4–17.0)	(22.9)	(12.5–33.2)	(19.5)	(15.3–23.6)	(11.1)	(3.1–19.1)	(6.3)	(0.0–13.4)	(32.0)	(16.4–47.7)	(19.5)	(16.1–23.0)
50–59	(20.4)	(19.0–21.8)	(20.2)	(9.3–31.0)	(13.7)	(10.5–17.0)	(8.6)	(1.0–16.2)	(29.5)	(14.7–44.4)	(9.7)	(2.7–16.6)	(14.7)	(11.9–17.5)
60–69	(18.2)	(16.8–19.5)	(6.4)	(1.1–11.8)	(6.0)	(3.9–8.0)	(7.4)	(0.9–13.9)	(21.1)	(9.7–32.6)	(7.4)	(0.7–14.1)	(7.2)	(5.4–9.1)
≥70	(12.0)	(10.9–13.1)	(0.7)	(0.0–2.1)	(1.4)	(0.4–2.4)	NA	NA	(5.3)	(0.0–11.4)	(3.4)	(0.0–7.2)	(1.6)	(0.8–2.5)
p-value				0.01§		<0.0001§		<0.0001§		0.20		<0.01§		<0.0001§
**Sex**														
Female	(50.2)	(48.2–52.1)	(73.3)	(61.9–84.7)	(60.8)	(55.1–66.5)	(54.6)	(36.3–73.0)	(57.9)	(40.4–75.4)	(50.7)	(34.2–67.2)	(60.7)	(56.0–65.3)
Male	(49.8)	(47.9–51.8)	(26.7)	(15.3–38.1)	(39.2)	(33.5–44.9)	(45.4)	(27.0–63.7)	(42.1)	(24.6–59.6)	(49.3)	(32.8–65.8)	(39.3)	(34.7–44.0)
p-value				<0.001§		<0.001§		0.64		0.40		0.95		<0.0001§
**Education**														
Less than high school	(12.7)	(11.1–14.2)	(9.9)	(0.0–20.6)	(7.5)	(3.7–11.3)	(10.6)	(0.0–24.6)	(13.7)	(0.0–30.8)	(30.5)	(13.0–48.0)	(10.5)	(6.8–14.2)
High school	(31.5)	(29.7–33.3)	(10.5)	(3.5–17.4)	(19.4)	(15.0–23.7)	(24.6)	(9.3–39.9)	(16.6)	(5.1–28.1)	(37.0)	(21.6–52.5)	(20.2)	(16.6–23.8)
Some college	(29.1)	(27.4–30.8)	(44.0)	(30.4–57.6)	(29.5)	(24.3–34.7)	(17.0)	(5.9–28.1)	(22.6)	(8.6–36.6)	(16.8)	(6.2–27.4)	(28.6)	(24.4–32.8)
Bachelor’s or higher	(26.7)	(25.1–28.4)	(35.6)	(23.5–47.8)	(43.7)	(38.0–49.3)	(47.8)	(29.4–66.2)	(47.1)	(29.8–64.3)	(15.7)	(6.3–25.0)	(40.7)	(36.2–45.2)
p-value				0.01§		<0.0001§		0.10		0.10		<0.01§		<0.0001§
**Race/Ethnicity**														
White, non-Hispanic	(66.4)	(64.4–68.4)	(65.4)	(51.6–79.2)	(67.5)	(61.7–73.4)	(45.2)	(27.6–62.8)	(71.2)	(54.2–88.3)	(53.3)	(36.6–70.0)	(64.5)	(59.6–69.3)
Hispanic	(14.8)	(13.2–16.4)	(18.3)	(7.5–29.0)	(14.9)	(10.2–19.6)	(15.9)	(1.2–30.6)	(18.0)	(1.9–34.1)	(18.7)	(3.9–33.5)	(15.9)	(12.0–19.8)
Black, non-Hispanic	(11.9)	(10.6–13.3)	(5.8)	(0.6–11.0)	(6.2)	(3.5–8.8)	(21.2)	(4.7–37.6)	(10.0)	(0.4–19.6)	(22.2)	(7.8–36.7)	(9.0)	(6.2–11.8)
Other, or ≥2 races	(6.8)	(5.6–8.0)	(10.5)	(0.0–22.3)	(11.4)	(7.0–15.9)	(17.7)	(1.3–34.1)	(0.8)	(0.0–2.4)	(5.7)	(0.0–13.7)	(10.6)	(7.0–14.2)
p-value				0.47		0.01§		0.06		0.48		0.28		0.04§
**Metropolitan living area**														
Metro	(83.7)	(82.2–85.1)	(84.8)	(75.9–93.8)	(87.7)	(84.2–91.2)	(87.3)	(77.2–97.4)	(88.1)	(77.5–98.8)	(85.1)	(72.9–97.2)	(87.1)	(84.2–90.0)
Nonmetro	(16.3)	(14.9–17.8)	(15.2)	(6.2–24.1)	(12.3)	(8.8–15.8)	(12.7)	(2.6–22.8)	(11.9)	(1.2–22.5)	(14.9)	(2.8–27.1)	(12.9)	(10.0–15.8)
p-value				0.81		0.05		0.53		0.48		0.83		0.05
**Region**														
Northeast	(18.1)	(16.6–19.6)	(24.9)	(13.2–36.7)	(17.6)	(13.5–21.6)	(32.6)	(13.9–51.4)	(5.1)	(0.0–12.4)	(8.1)	(0.3–15.9)	(17.9)	(14.4–21.5)
Midwest	(21.1)	(19.6–22.6)	(21.6)	(11.3–31.8)	(23.6)	(19.0–28.2)	(13.6)	(3.4–23.8)	(35.6)	(19.7–51.4)	(17.3)	(5.1–29.5)	(22.8)	(19.1–26.5)
South	(37.2)	(35.3–39.1)	(34.0)	(20.9–47.1)	(34.5)	(28.9–40.0)	(41.8)	(24.5–59.2)	(34.0)	(16.0–51.9)	(57.5)	(41.3–73.7)	(37.1)	(32.5–41.7)
West	(23.6)	(21.9–25.3)	(19.5)	(8.6–30.4)	(24.3)	(19.2–29.5)	(11.9)	(0.0–25.9)	(25.4)	(11.0–39.7)	(17.1)	(4.3–29.9)	(22.2)	(18.1–26.3)
p-value				0.60		0.70		0.13		0.10		0.07		0.85

**Abbreviations:** CI = confidence interval; NA = not available (insufficient sample size).

* Based on responses to Porter Novelli 2014 summer ConsumerStyles survey with questions on contact lens use and wearer/non-wearer demographics as of summer 2014.

† Other = Contact lens wearers that said they wore another type of contact lens not captured by the survey choices.

§ Significantly different from non-wearers at the 95% confidence level.

**TABLE 2:** Prevalence of risk behaviors for eye infections* among contact lens wearers, stratified by type of contact lens — United States, 2014

Risk factor/Behavior	% of wearers, by type of contact lens				

	Daily disposable (n = 154)	Planned replacement, soft (n = 730)	Overnight, soft† (n = 182)	Rigid (n = 85)	Overall (n = 1,141)
**Sleeping overnight in contact lens (ever)** §	(48.7)	(45.1)	(88.6)	(17.3)	(50.2)
**Napping in contact lens (ever)**	(85.1)	(86.9)	(96.4)	(74.1)	(87.1)
**Topping off solution (ever)**	(72.0)	(51.3)	(59.3)	(60.5)	(55.1)
**Replacing lenses at interval longer than recommended or when problem**	(39.0)	(48.5)	(47.4)	NA¶	(49.9)
**Not using contact lens case**	(39.6)	(1.9)	(13.4)	(0.0)	(8.9)
**Replacing contact lens case at interval longer than recommended**	(83.9)**	(81.1)	(82.0)	(91.4)	(82.3)
**Storing lenses in tap water (ever)**	(28.0)**	(12.4)	(20.9)	(33.3)	(16.8)
**Rinsing lenses in tap water (ever)**	(40.3)	(27.2)	(38.3)	(91.4)	(35.5)
**Showering in contact lens (ever)**	(85.1)	(84.6)	(94.6)	(67.5)	(84.9)
**Swimming in contact lens (ever)**	(59.1)	(61.7)	(64.9)	(50.6)	(61.0)
**Infrequently or never washing hands before inserting lenses**	(1.3)	(4.8)	(2.4)	(2.5)	(3.7)
**Infrequently or never washing hands before removing lenses**	(19.5)	(12.5)	(9.0)	(17.3)	(13.3)
**Where lenses were purchased**					
Provider office	(66.9)	(64.7)	(67.5)	(84.0)	(66.9)
Retail store without eye exam	(8.4)	(11.8)	(7.5)	(8.6)	(10.4)
Internet	(23.4)	(21.3)	(24.4)	(4.9)	(20.8)
**Had a red/painful eye while wearing contact lens that required a doctor’s visit (ever)**	(29.2)	(29.3)	(35.3)	(28.9)	(30.2)

* Based on responses to Contact Lens Risk Survey, reflecting usual behaviors as assessed in December 2014.

† Overnight contact lens wearers replied “yes” to “Are your contact lenses recommended by your eye doctor for overnight wear?”

§ Ever indicates the combined results of those who answered question “always,” “fairly often,” “sometimes,” or “infrequently” (i.e., questions with these responses were coded as “ever” if the response was not “never”).

¶ NA = 100% of rigid wearers reported replacing their lenses when they had a problem, which is compliant with recommendations for rigid lenses.

** Case replacement and storage in tap water questions were only asked if respondent reported using a contact lens case; 39.6% of daily disposable wearers did not use a case. Thus, the reported percentages are the proportion of the 60.4% (n = 93) of daily disposable users that reported using a case.

## Footnotes

* Porter Novelli Public Services. ConsumerStyles 2014 Methodology. Washington, DC: Deanne Weber; 2014.

† Adapted from Wagner H, Richdale K, Mitchell GL, et al. Age, behavior, environment, and health factors in the soft contact lens risk survey. Optom Vis Sci 2014;91:252–61. Responses from the Contact Lens Risk Survey reported here reflect usual behavior as assessed in December 2014.

§ Based on 16.7% of respondents who reported wearing contact lens and U.S. Census Bureau population estimate of population aged ≥18 years on June 1, 2014. Available at http://www.census.gov/popest/data/national/asrh/2014/index.html.

¶ In the NHANES protocol, a contact lens wearer was defined as a study participant wearing contact lenses at the time of their examination and who used contact lenses for distance vision.

* Adapted from previously published information: Collier SA, Gronostaj MP, MacGurn AK, et al. Estimated burden of keratitis—United States, 2010. MMWR Morb Mortal Wkly Rep 2014;63:1027–30.

† These recommendations were developed through solicitation of expert consensus opinion and scientific literature review by CDC in collaboration with a workgroup that included members from the U.S. Food and Drug Administration, the American Academy of Ophthalmology, the American Academy of Optometry, the American Optometric Association, the Contact Lens Association of Ophthalmologists, the Contact Lens Society of America, and the National Academy of Opticianry. The rationale and publications used to support these recommendations can be found on CDC’s Healthy Contact Lens “Show Me the Science” web page, available at http://www.cdc.gov/contactlenses/show-me-the-science.html.
